# Global public health leadership: The vital element in managing global health crises

**DOI:** 10.7189/jogh.12.03003

**Published:** 2022-02-05

**Authors:** Krzysztof Goniewicz, Frederick M Burkle, Thomas Falconer Hall, Mariusz Goniewicz, Amir Khorram-Manesh

**Affiliations:** 1 Department of Security Studies, Military University of Aviation, Dęblin, Poland; 2 Harvard Humanitarian Initiative, T.H. Chan School of Public Health, Harvard University, Boston, Massachusetts, USA; 3 AMS Support Unit, Army Medical Services, Camberley, United Kingdom; 4 Department of Emergency Medicine, Interfaculty Centre for Didactics, Medical University of Lublin, Lublin, Poland; 5 Institute of Clinical Sciences, Department of Surgery, Sahlgrenska Academy, Gothenburg University, Gothenburg, Sweden; 6 Gothenburg Emergency Medicine Research Group (GEMREG), Sahlgrenska Academy, Gothenburg University, Gothenburg, Sweden

The World Health Organization (WHO) and the International Health Regulations Treaty (IHRT) are responsible for modelling global public health crises, and management and mitigation of their consequences. However, both duties are delivered in all nations by their national public health systems. Therefore, the implementation of public health policies at the national level depends on the public trust of the national authorities. A trustful relationship is necessary for developing and maintaining the well-being of a community through various public health programs [1]. The principle aim of public health programs is to assess all risks, to identify underserved populations, and to initiate preventive measures, such as vaccines, non-pharmaceutical interventions (eg, social distancing, isolation) and vector control, through collaboration and coordination with other agencies and organizations, such as hospitals, schools [1]. These efforts require management authority, resources and financial support for public health and community research and sustainability of the changes they demand [1] **(****Figure 1****).**

**Figure 1:**
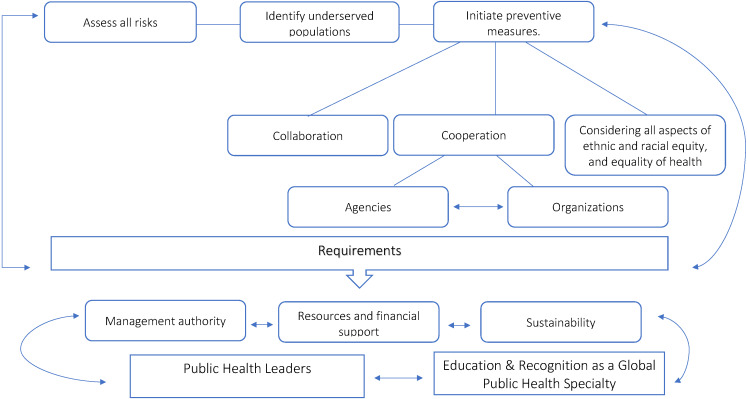
Aim of public health programs

WHO declared early in the Covid-19 pandemic that “there's no going back to normal.” This was a clear message that the existing public health infrastructure and response, seen as the “difference between life and death,” was “inadequate for the impending crisis.”

Public Health management of infectious disease outbreaks and epidemics is complex and often politically heralded in the media for a short while, with the actual voices and advocates of success largely going unnoticed. Too often, public health suffers from being a victim of its own successes, with many unaware that those major public health challenges ever existed [2]. Paradoxically, public health leaders and advocates are now facing a global pandemic with the world watching, judging, and criticizing their every move [2]. This report highlights the significant efforts that require management authority, resources and financial support for public health and community resources research and sustainability of the changes they demand.

## SUCCESSFUL IMPLEMENTATION OF PUBLIC HEALTH

The key to success in the face of a public health crisis lies in prevention, preparedness, communication, and control of infectious and environmental diseases. Often called the “invisible health profession”, public health specialty is largely responsible for the majority of “improvements in global health expectancy” [2]. Developed countries expect public health to play a significant role in managing outbreaks of infectious diseases and epidemics, and overall, it has performed well. Adequate epidemiological analysis and effective preventive measures have not overwhelmed the global healthcare system allowing timely treatment of patients. As the prevalence of some diseases increase, health promotion strategies bring more resources and financial support to both local and global health resources. This was clearly evident in epidemic outbreaks of H1N1, SARS, and Ebola, to name but a few. Societies rely on public health professionals who know how to utilize resources efficiently, creating, organizing, and implementing a variety of public health strategies and programs for the benefit of the global population [3].

Public health aims to serve the whole population, recognizing that populations are not homogenous entities. Factors such as sex, race, disability, migration status and socioeconomic position can intersect to result in certain groups in society being particularly at-risk during health crises [2]. Indeed, population-based management, not individual one-on-one care, is the mainstay of public health management and success. COVID-19 pandemic has resulted in disparities in health outcomes, with the most vulnerable being disproportionately impacted throughout the pandemic [4]. Therefore, public health preparedness plans must consider the impact of a health crisis on the whole population and include underserved, vulnerable or stigmatized groups (such as undocumented migrants) [4]. The rationale for this inclusion is for both justice and enlightened self-interest; to reduce the exacerbation of existing health disparities and to optimize the collective emergency response.

The field of public health offers a multiagency and multi-professional collaboration that teaches openness and readiness for changes and challenges in the course of a disease. A public health specialist can identify threats to the health of the population, that is, threats beyond the disease of the individual patient alone [5]. Epidemiology enables the analysis of the prevalence of diseases in society and the study of the factors that influence their emergence. The most important features of a public health specialist are the willingness to search for the causes and origin of diseases and selecting appropriate tools for various tasks, communication, and cooperation in population-based teams [3]. There is a need for efficient coordination of pro-population-based health activities to prevent the consequences of disease or at least mitigate their unwanted outcomes. That is why unfairly, public health has often been labelled as a passive specialty focusing only on preventable diseases [6].

The ongoing pandemic of COVID-19 has emphasized the significance of public health as a unique specialty and its leading role in emerging public health emergencies [5]. It has also shown the importance of treating the origin of such a tumour on society and not only its metastasis. 2020 has proven to be a challenging year for public health leaders who had to go through an unimaginable crisis for which many health care systems were not prepared [7]. Collaborative efforts are crucial in managing public health crises. Epidemic control demands a response that outstrips the capacity and authority of any single organization; public health leaders, often in positions without direct authority, must therefore be skilled in influencing a coherent multi-stakeholder response [8].

In the United States, both economic and political leaders at state and national levels subsumed the leadership of public health decisions from the start, with many public health experts summarily being ignored, dismissed, threatened, or completely disregarded [9]. One in eight Americans - roughly 40 million people - lives in a community that has lost its local public health department leader during the coronavirus pandemic, all because of lack of political support or controlling political interference that prevented these leaders from making unpopular but necessary public health decisions [1]. Collectively, public health experts say the loss of expertise and experience has created a leadership vacuum in the profession [3].

**Figure:**
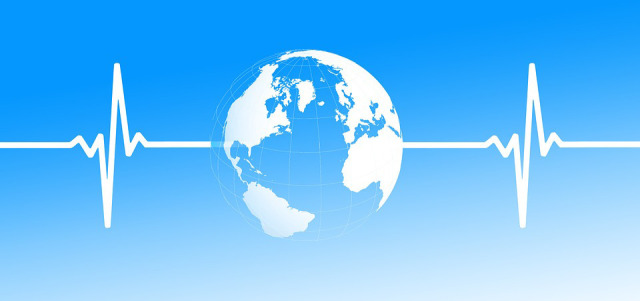
Photo: From https://pixabay.com/illustrations/globe-earth-heart-rate-pulse-762008/.

Public trust in public health agencies is an essential prerequisite for a successful response to a health crisis. Trust is required for motivating the public to undertake both voluntary action (such as vaccination uptake [10]) or to maintain compliance with legally binding regulations (such as stay-at-home orders), recognizing that government policies in free societies require the consent of the governed [11]. Public health agencies must not succumb to naive utilitarianism and instead consider the long-term consequences of individual policies on trust in the overall public health response [10,11]. Trustworthy public health messages are required to be scientifically based and non-political, a challenging task given inherent scientific uncertainty during health crises (which can result in policy inconsistencies) and the requirement for public health officials to work closely with politicians to deliver an effective response [11].

If there is one universal lesson that this pandemic teaches us is that a new generation of public health leadership and authority, better trained, respected, and managed, will have to be established in most communities worldwide and under a strong and independent WHO.

Resolute public health leadership at global, national and local levels has decisively influenced crisis response. At the global level, Gro Harlem Brundtland’s leadership of the WHO during SARS was characterized by swiftly galvanizing the international community to action and honestly engaging with (and at times challenging) national governments to contain the outbreak [12]. At the national level, Nigeria’s response to Ebola in 2014 saw strong political and public health leadership build on a pre-existing commitment to strengthening public health capacity to rapidly contain the outbreak, which resulted in only twenty cases and eight deaths [13]. At the local level, insight from three UK case studies identified factors grouped into “*getting started*”, “*maintaining momentum*” and “*indicators of success*” themes that contribute to success in systems leadership [14]. One of these cases was the 2018 Salisbury Novichok poisoning response. The challenges of leading this incident’s response have recently been given a wider audience through the BBC’s television drama *The Salisbury Poisonings*, resulting in media interest in the complexities of public health decision-making and communication [15].

On the other hand, it must be remembered that there have been cases where politicians have taken the role of scientific experts and give recommendations on which vaccines are “good” and which ones should not be taken [16]. National public health responses should outline a clear delineation between the roles and responsibilities of political leaders, who have the legitimacy and authority to lead the policy response and health officials who give medical and technical advice to officials and the public, such as the UK’s Joint Committee on Vaccination and Immunisation.

Public health emergencies will become more frequent in the future with accelerating climate changes, rapid urbanization, scarcities in food, water and energy resources, and deforestation to name but a few [3]. Public health leadership must focus on improving communication and leadership skills [6]. It cannot afford to be a passive behind the scenes profession. This must be reflected in improved quality of data, data analysis, forecasting, infrastructure improvements, a strong focus on mitigating economic and social (including racial) community-wide inequalities and improved community resilience to help public health leaders successfully move through all phases of future pandemic and global public health challenges [2].

## RECOMMENDATIONS

Several measures should be implemented immediately, such as alternative local leadership and alternative facilities for care to facilitate a bid for a more flexible surge capacity to increase the four essential elements of surge capacity, ie, staff, stuff, structure, and systems [3]. A major role for Public Health and public education is a critical ingredient of a flexible response system in an age of increasing weather/climate disasters and epidemic and pandemic risks. One of the main obstacles to implementing public health policies and strategies is the low prevalence of health literacy globally. Several reports from the US, Southeast Asian countries and Europe have indicated a low or limited global health literacy, resulting in worse health care and poorer health outcomes [17]. This health literacy is consistently associated with several factors such as education, ethnicity, and age. The lack of necessary skills required to understand and manage their health does not allow the active participation of vulnerable groups in disease prevention programs [18]. Having a sustainable program guarantees public health’s continuous use to achieve its aims and population outcomes. To achieve such sustainability, public health leaders need to have the authority to evaluate whether a program is continued or halted for any reason. They also need to create systems and control measures to evaluate the benefits or outcomes of a program for its consumers. The latter requires good collaboration with the social and community-related organizations before, during and after program implementation. A course of sustained collaboration may also identify other sites in need of immediate improvement and where the program, sensitive to environmental, cultural, and socioeconomic demands, must be replicated to guarantee the long-term outcomes of the entire global program [3].

Lastly, population-based management of health crises must become a recognized health specialty which has as its core the training of future Health Crisis Managers and scientists trained across the entire “disaster cycle’ not just that of the “response phase” alone. Such recognized experts, all with a core of public health training and expertise, would become the population-based managers serving every WHO-recognized community level program [19].

## CONCLUSIONS

Global crisis management demands responsible public health leadership, receptive to well-established experiences and new suggestions and exercising vocal anti-discrimination policies effectively [20]. Public health schools worldwide have to equip their students with the tools and resources they need to become effective leaders in uncertain times. They must look to not only train the best epidemiologists and biostatisticians, but also equip students with the skills and expertise to discharge public health leadership roles within the political environment. These important steps must be taken to guarantee a better future for global health and offer opportunities and new insights to health care leadership in a flexible response system [8]. With improvements in the inherent knowledge, innovation, education, and support from all emergency and non-emergency organizations, public health must be the leading specialty to build upon and create a new culture of safety and resilience at all levels of society measured by innovative priorities for action in disaster risk reduction and response [3]. If the world was able to develop global communications and air travel, it should also be eager and able to develop what we now appreciate as the future of “global public health” [9].
